# Whole genome characterization of Torque teno sus virus 1 (TTSuV1) in wild and domestic pigs: insights into genetic classification, host differentiation, and intra-host variation

**DOI:** 10.3389/fmicb.2025.1585558

**Published:** 2025-05-09

**Authors:** Xiaolong Li, Yasmin Tavares, Céline M. Carneiro, Caroline Phillips, Kuttichantran Subramaniam, John Lednicky, Raoul K. Boughton, Kim M. Pepin, Ryan S. Miller, Kurt C. VerCauteren, Samantha M. Wisely

**Affiliations:** ^1^Department of Wildlife Ecology and Conservation, University of Florida, Gainesville, FL, United States; ^2^Department of Infectious Diseases and Immunology, College of Veterinary Medicine, University of Florida, Gainesville, FL, United States; ^3^Emerging Pathogens Institute, University of Florida, Gainesville, FL, United States; ^4^Department of Environmental and Global Health, College of Public Health and Health Professions, University of Florida, Gainesville, FL, United States; ^5^Buck Island Ranch, Archbold Biological Station, Lake Placid, FL, United States; ^6^National Wildlife Research Center, United States Department of Agriculture, Animal and Plant Health Inspection Service, Wildlife Services, Fort Collins, CO, United States; ^7^Center for Epidemiology and Animal Health, United States Department of Agriculture, Animal and Plant Health Inspection Service, Veterinary Services, Fort Collins, CO, United States

**Keywords:** Torque teno sus virus 1, wild pigs, genetic diversity, host-specific differentiation, intra-host variation

## Abstract

**Background:**

Torque teno sus virus 1 (TTSuV1), a member of the *Anelloviridae* family, is highly prevalent in swine populations and exhibits substantial genetic diversity. Despite its ubiquity, TTSuV1 remains understudied, particularly regarding its genetic diversity, host-specific differentiation, and intra-host variation. These characteristics are critical for understanding its evolution, transmission dynamics, and potential applications in biosecurity monitoring.

**Methods:**

Field and laboratory protocols included capturing wild pigs, collecting whole blood samples, and screening for TTSuV1-positive samples through PCR. TOPO TA cloning was used to amplify individual viral variants within hosts, and whole genome sequencing (WGS) was performed on selected clones. A dated phylogenetic tree was reconstructed using TTSuV1 whole genome sequences obtained from wild pig samples in this study and all available sequences from NCBI. To evaluate genetic differentiation between wild and domestic pigs, partial viral sequences (~700 bp) were analyzed using phylogenetic D statistic and analysis of molecular variance (AMOVA). Intra-host variation was assessed by calculating pairwise identity percentages among viral clones from individual hosts and constructing haplotype networks.

**Results:**

Phylogenetic analysis of whole genome sequences grouped TTSuV1 into four clades, with sequences from wild pigs distributed across all clades. Known subtypes 1a, 1b, and 1c were localized within Clades 3 and 4, leaving sequences in Clades 1 and 2 with unidentified subtypes. Partial sequence analysis revealed significant host-specific genetic differentiation: the D statistic confirmed a non-random association between host type (wild vs. domestic) and phylogeny, and AMOVA further showed contributions of both host type and geography to overall variation. Intra-host variation analysis provided evidence for multiple sources of genetic diversity within individual hosts. Pairwise identity percentages among viral clones ranged from 63.6% to 100%, with lower identity values indicating co-infection with distinct viral variants. Haplotype network analysis revealed mutational steps between haplotypes from the same host, suggesting that intra-host evolution also contributes to within-host genetic variation.

**Conclusions:**

This study highlights the significant genetic diversity and host-specific differentiation of TTSuV1, with wild pigs playing a key role in its evolution. Both intra-host evolution and co-infection contribute to its diversity, underscoring its potential as a tool for monitoring biosecurity risks and cross-transmission between wild and domestic pigs.

## 1 Introduction

Torque teno sus viruses (TTSuVs) are small, circular, single-stranded DNA viruses that infect domestic and wild pigs (*Sus scrofa*) and belong to the *Anelloviridae* family (Webb et al., 2020). Two types of TTSuV have been identified: Torque teno sus virus 1 (TTSuV1) and Torque teno sus virus 2 (TTSuV2). To date, studies have consistently found the virus to be present across all populations of both domestic and wild pigs examined, suggesting endemicity, with reported prevalence ranging from 46% to 94% in domestic pigs and 32% to 66% in wild pigs worldwide (Aramouni et al., 2011; Bigarr et al., 2005; Cadar et al., 2013; Martínez et al., 2006; Cortey et al., 2012). Although TTSuVs are generally considered non-pathogenic when acting alone, co-infection with these viruses may modulate host immune responses, potentially exacerbating the severity of other viral or bacterial infections (Mei et al., 2011; Krakowka and Ellis, 2008; Ellis et al., 2008). Despite their widespread presence, the biology and transmission dynamics of TTSuVs remain poorly understood, and their diversity and evolutionary dynamics are still underexplored.

TTSuV's persistence and transmission dynamics, combined with its ubiquitous presence, make them an intriguing subject for virological research in swine populations (Li et al., 2024). Wild pigs (either native European boar or invasive wild pigs) have been identified as reservoirs for various pathogens and are a recognized source of transboundary animal diseases that threaten domestic pig populations (Miller et al., 2017; Keiter et al., 2016). Their potential to introduce novel pathogens into domestic pig herds presents a significant biosecurity risk for pig farms. Studies of endemic TTSuV in wild and domestic populations could potentially provide valuable information about biosecurity breaches or inferences about other potential pathogen transmission dynamics. Despite this potential, very little is known about the similarities or differences between TTSuVs circulating among or between wild and domestic pigs. Addressing these knowledge gaps could provide novel insights into host-specific viral evolution and the mechanisms driving transmission between these populations.

Among the two TTSuV species, TTSuV1 has been found to exhibit greater genetic diversity compared to TTSuV2 (Cortey et al., 2011). This higher level of genetic diversity enhances TTSuV1's suitability for molecular studies investigating viral evolution and transmission dynamics (Li et al., 2024). For these reasons, this paper focuses specifically on TTSuV1. Currently, the International Committee on Taxonomy of Viruses (ICTV) recognizes only two subtypes of TTSuV1 (a and b); additional subtypes (c, d, etc.) have been proposed in previous studies based on partial genomic sequences (Webb et al., 2020; Cortey et al., 2011; Huang et al., 2010; Liu et al., 2013). However, because these classifications are based on incomplete genomic information their accuracy and utility for understanding TTSuV1 diversity and its implications for viral evolution may be somewhat limited. To date, no comprehensive study has utilized whole genome sequencing (WGS) data to refine the genetic classification for TTSuV1, which creates a foundational gap in our understanding of the viral diversity. A WGS-based approach is essential for advancing molecular epidemiological studies of TTSuV1 to unveil its transmission dynamics in swine populations.

Another significant gap lies in the limited information regarding TTSuV1 genetic differentiation between wild and domestic pig populations. Current literature includes very few TTSuV1 sequences isolated from wild pigs, with nearly no whole genome sequences available from this group. This lack of WGS data restricts our ability to understand potential genetic divergences between TTSuV1 populations infecting wild versus domestic pigs. Such differentiation could reveal potential cross-population transmission events (Rudova et al., 2022). Identifying clear genetic distinctions would allow us to monitor biosecurity risks posed by wild pig populations and aid in detecting potential biosecurity breaches where wild and domestic pig populations interact, with significant implications for disease control and farm management.

In addition to high between-host genetic variation, TTSuV1 also exhibits high variation within individual hosts (Li et al., 2019). However, the origins of intra-host genetic variation in TTSuV1 remain poorly understood. Mutations during replication, co-infection with different viral strains, recombination events, and selective pressures imposed by the host environment are thought to be common drivers of intra-host variation (Leigh et al., 2021; Wu et al., 2024; Pipek et al., 2024). Analyzing the sources of intra-host variation of TTSuV1 could provide critical insights into the mechanisms by which TTSuV1 adapts to host immune pressures, maintains persistent infections within individual hosts, and spreads across host populations (Ko et al., 2024; Landis et al., 2023; Sun et al., 2023; Wang et al., 2021), which is essential for gaining a comprehensive understanding of how TTSuV1 evolves over time. Moreover, this knowledge not only informs our broader understanding of TTSuV1's evolutionary dynamics but also sheds light on its potential role in shaping viral transmission patterns and influencing interactions with other pathogens in co-infections.

In this study, we aim to address these critical knowledge gaps through comprehensive whole genome analyses, contributing to the understanding of TTSuV1 genetic classification, host-related genetic differentiation, and intra-host variation. Specifically, the objectives of this paper are:

1. **To provide insights into the diversity of TTSuV1 genomes relative to current genetic classification** by using WGS data from both our samples and the National Center for Biotechnology Information (NCBI) records. This evaluation of diversity will offer a more comprehensive view of TTSuV1's genetic variability.
2. **To examine the genetic differentiation of TTSuV1 between wild and domestic pig populations** by analyzing TTSuV1 genomes from wild and domestic pigs. We hypothesize that TTSuV1 isolates from wild and domestic pigs will show clear phylogenetic separation, indicating host-specific viral evolution. Distinct clustering among phylogenetic tree branch tips would suggest that the origin of a TTSuV infection could be inferred and that evidence of mixed wild and domestic TTSuV1 lineages within a domestic population could point to potential biosecurity breaches in pig farms.
3. **To investigate sources of intra-host variation of TTSuV1 by focusing on viral clones within different hosts**. We will identify the multiple drivers of intra-host diversity to enhance our understanding of TTSuV1's evolutionary dynamics.

## 2 Materials and methods

### 2.1 Field and laboratory protocols

Wild pigs were live captured using baited traps at Archbold's Buck Island Ranch (ABIR; Lake Placid, Florida, USA) from February 2017 to February 2018. We chemically immobilized wild pigs at the point of capture using a cocktail of Telazol (50 mg/ml, Zoestis, Parsippany, NJ, USA) and Xylazine (100 mg/ml, Akorn Inc., Lake Forest, IL, USA) at a doseage of 4.4 mg/kg for Telazol and 2.5 mg/kg of Xylazine under the approved University of Florida Institutional Animal Care and Use Committee (IACUC) Protocol 201808495. Whole blood samples (0.5 to 1 mL stored in ethylenediaminetetraacetic acid [EDTA] or mammalian lysis buffer) were collected from wild pigs via venipuncture of the marginal ear vein and stored at −20°C until extracted. After sample collection, we ear-tagged animals and released them at the point of capture after full recovery from anesthesia.

Polymerase chain reaction (PCR) was first used to screen for TTSuV1-positive samples. Total DNA was extracted from whole blood samples using the Gentra Puregene Blood Kit (Qiagen, Germantown, USA) following the manufacturer's protocol for compromised blood. When EDTA-treated blood was unavailable for a given animal, whole blood in mammalian lysis buffer was used for screening purposes. DNA concentrations were measured with a Nanodrop One spectrophotometer (Thermo Fisher Scientific, Waltham, USA), and any samples exceeding 100 ng/μl were diluted to 75 ng/μl to minimize the risk of PCR inhibition. A one-step PCR assay, utilizing previously described primers, was performed to detect TTSuV1 based on a 678-base pair (bp) region that includes parts of the untranslated region (UTR) and open reading frame (ORF) 1, as well as the entire ORF2 (Cortey et al., 2012). PCR products were analyzed on 2% agarose gels to confirm the presence of amplicons as expected for TTSuV1-positive samples.

To amplify the entire genome (~ 2.8 kb) of TTSuV1, positive samples were reamplified using primers PTTV1F2 and PTTV1R2 described in the literature (Liu et al., 2013). In a 50 μl reaction, we placed 10 μl of template DNA, 0.3 μM of each primer, 400 μM of dNTP's, 5 μl of Takara LA Taq buffer without Mg^2+^, 3 mM MgCl_2_, and 2.5 U of Takara LA Taq enzyme (Takara Bio USA, San Jose, California). This product is a high-fidelity, long-read DNA polymerase with a DNA proofreading polymerase for the purpose of identifying small variations among sequences. Reaction conditions began with a 94°C denaturation for 1 min followed by 35 cycles of 94°C for 2 min, 62°C for 30 s, 72°C for 3 min, and a final extension at 72°C for 10 min. All products were purified using a Wizard SV Gel and PCR clean-up kit (Promega, Madison, WI, USA).

To facilitate the whole genome sequencing of multiple viral variants of TTSuV1 from a single host, we employed TOPO TA cloning to amplify individual viral variants within a host. TOPO TA cloning was suitable for the whole genome because the TTSuV1 genome is ~2.8 kb in length. This method provided a straightforward and efficient way to capture PCR-amplified DNA variants from a single host for subsequent propagation, sequencing, and analysis. With the Invitrogen TOPO TA Cloning Kit (Invitrogen, Waltham, MA, USA), whole genome PCR products were inserted into the plasmid vector, and the recombinant plasmids were transformed into *E. coli* using the manufacturer's protocol. Up to five bacterial colonies containing transformed plasmids were grown overnight (16–20 h) in LB broth at 37°C and 200 rpm prior to plasmid purification using a QIAprep Spin Miniprep Kit (Qiagen, Germantown, USA).

### 2.2 Whole genome sequencing and genome assembly

Purified plasmid DNA was used to generate DNA sequencing libraries with the NEBNext Ultra II DNA Library Prep Kit (New England Biolabs, Ipswich, MA, USA) following the manufacturer's instructions. The resulting libraries were then sequenced using a v3 chemistry 600 cycle kit on a MiSeq platform (Illumina, San Diego, CA, USA). Before genome assembly, raw sequencing reads were assessed for quality using FastQC (v0.11.9). Adapter sequences and low-quality bases (Phred score < 20) were trimmed using Trimmomatic (v0.39). We then performed *de novo* genome assembly using SPAdes (v3.15.3) with default parameters, except for setting the coverage cutoff to 100 to exclude low-confidence regions. This approach ensured a robust assembly of high-confidence contigs suitable for downstream analyses. The resulting contigs were assessed for quality using QUAST (v5.0.2) and were confirmed by BLAST searches against known TTSuV1 references.

### 2.3 Evaluating diversity of TTSuV1 genomes

To better understand the evolution and diversity of TTSuV1 at the whole genome level, a time-dated phylogenetic tree was reconstructed using whole genome sequences of the virus obtained from the wild pig samples in this study and all available whole genome sequences of the virus downloaded from NCBI. We used the following query terms to search at NCBI: (“Torque teno sus virus 1a” OR “Torque teno sus virus 1b”) AND (“complete genome”). To further filter out any potential TTSuV2 genome sequences in the downloaded dataset, a neighbor-joining tree was first built in MEGA version 11 (Tamura et al., 2021) to differentiate these two types of TTSuV. TTSuV2 genome sequences were then removed from the downloaded dataset, and the rest of the sequences were included into the time-dated tree reconstruction. Additionally, host type information (domestic or wild pig) was extracted from the metadata of the NCBI entries for further analysis. In this study, host type referred specifically to wild versus domestic (farmed) pigs. Due to limitations in available metadata, information such as age, sex, and herd composition was not consistently reported and was therefore not included in the analysis

Whole genome sequences were first aligned using the MUSCLE module embedded in Geneious Prime 2023.1.2 (https://www.geneious.com) and then used to reconstruct a time-dated phylogenetic tree in the BEAST 2 software package (Bouckaert et al., 2014). The optimal substitution model was identified with MEGA version 11 to ensure accurate phylogenetic inference. The Coalescent Bayesian skyline model and an optimized relaxed clock model were chosen as the population and molecular clock models, respectively. A prior for the mean clock rate of 5.0 × 10^−4^ substitutions per site per year was applied, based on previous estimates (Cadar et al., 2013). To ensure sufficient mixing, we employed a Markov chain Monte Carlo (MCMC) chain length of 200,000,000 iterations, with parameter convergence verified in Tracer version 1.7.2, requiring an effective sample size (ESS) >200. Finally, TreeAnnotator version 2.7.5 was used to construct the maximum clade credibility (MCC) tree with a 10% burn-in.

### 2.4 Examining genetic differentiation of TTSuV1 between host types

Due to the absence of whole genome sequences of TTSuV1 from wild pig samples in NCBI, as well as to minimize sampling bias introduced by the whole genome sequences generated from wild pigs in this study, we utilized partial viral sequences (~700 bp) to investigate the genetic differentiation of TTSuV1 between wild and domestic pigs. This partial sequence covers part of the untranslated region (UTR) and open reading frame (ORF) 1 and the entire ORF2 of the TTSuV1 genome and has been commonly used in genetic studies of this virus (Cortey et al., 2012). To increase the number of viral sequences from wild pigs in our dataset, we first performed a literature search in PubMed using the following strategy: (torque teno sus virus) AND wild AND (pig OR hog OR boar). Wild pig-associated TTSuV1 sequences reported in the literature were then downloaded from NCBI. TTSuV1 sequences derived from domestic pigs in the same region/country where wild pig TTSuV1 samples were collected were also retrieved to create a dataset of wild and domestic pig TTSuV1 virus sequences that were paired by region/country. We aligned these downloaded partial sequences with the TTSuV1 whole genome sequences mentioned above and trimmed all sequences to the length of ~ 700 bp in Geneious Prime 2023.1.2 (https://www.geneious.com). A time-dated phylogenetic tree was reconstructed using the BEAST 2 software package, as described in the previous section.

Phylogenetic comparative methods (PCMs) are a collection of statistical methods that allow the exploration of associations between traits (e.g., host type) and evolutionary relationships by assessing whether related taxa share similar traits due to their common ancestry (Pennell and Harmon, 2013). To evaluate the relationship between TTSuV1 host type (wild or domestic pig) and the phylogenetic structure, we calculated the *D* statistic, which quantifies phylogenetic signal—a measure of how closely related viral sequences cluster by trait (host type), using the phylo.d function in R package “caper” (Fritz and Purvis, 2010). A *D* statistic value closer to 0 indicates strong phylogenetic clustering of traits, as expected under a Brownian motion model of evolution, while values near 1 indicating a random distribution of traits across the tree. Permutation tests were performed to assess the significance of the association between host type and phylogenetic structure.

To further examine genetic differentiation between host types, an analysis of molecular variance (AMOVA) was conducted. AMOVA is a simple but powerful statistical tool for analyzing genetic differentiation, as it partitions genetic variation into components attributable to differences among groups (e.g., host types or populations) and within groups (Excoffier et al., 1992). This analysis was used to identify whether genetic differentiation between wild and domestic pigs was statistically significant, considering both host type and geographic distribution (e.g., country). Given that wild pig populations from the USA and Europe are biogeographically distinct, and our primary focus was on differentiation between host types (wild vs. domestic), AMOVA was an appropriate approach to account for potential population differences among countries while assessing host-type-specific genetic variation. For this study, AMOVA was performed using the ‘poppr' package in R (Kamvar et al., 2015), which allows the integration of phylogenetic and genetic diversity data. Although TTSuV1 has been reported in pigs from many countries worldwide, only four countries (USA, Spain, Romania, and Uruguay) had publicly available sequences from both domestic and wild pigs in GenBank. Therefore, we limited our analyses to these countries to enable direct comparisons between host types (domestic and wild). Genetic variation was partitioned first among countries and then between host types to evaluate their contributions to overall genetic differentiation. The significance of genetic differentiation was tested using permutation tests.

### 2.5 Intra-host variation analysis

To investigate intra-host variation of TTSuV1 based on whole genome sequences, analyses were conducted on pig individuals with more than one viral clone in our dataset. Whole genome sequences of viral clones from selected individuals were aligned using the MUSCLE module embedded in Geneious Prime 2023.1.2 (https://www.geneious.com). Then, pairwise identity percentages were calculated for each individual as a metric to measure genetic similarity among viral clones within the same host.

Haplotype networks were constructed to visualize the genetic diversity of TTSuV1 within individual hosts. These networks are graphical representations where each node corresponds to a unique haplotype (genetic variant), and the connections between nodes represent mutational steps, defined as the number of nucleotide differences between haplotypes. Haplotype networks are particularly valuable for analyzing closely related sequences, as they provide a clear visualization of how haplotypes are connected through mutation. In this study, haplotype networks were generated for unique viral sequences found in host individuals with more than two unique clones using R package “pegas” (Paradis, 2010), based on aligned viral clone sequences. These networks illustrated the relationships among haplotypes within a host and provided insights into the processes shaping intra-host variation, such as mutation and recombination.

## 3 Results

### 3.1 Clones and whole genomes of TTSuV1 in this study

We screened a total of 183 wild pig samples collected between 2017 and 2018 in Florida, USA, and 58 individuals were PCR-positive for TTSuV1. Through the TOPO TA cloning procedure, 197 clones were generated from these 58 individuals and were whole genome sequenced afterwards. From these, 48 whole genome sequences of TTSuV1 were successfully assembled and used for the analyses (Supplementary Table S1). These whole genome sequences were derived from 22 wild pigs, of which 14 individuals had more than one clone, and 8 had more than two clones.

### 3.2 Phylogenetic analysis of TTSuV1 whole genome sequences globally

Through the initial search in NCBI, 127 TTSuV whole genome sequences were returned and downloaded. After the filtering process, 63 TTSuV2 whole genome sequences were removed, and the remaining 64 TTSuV1 whole genome sequences, along with the 48 sequences obtained in this study, were used for time-dated phylogenetic tree reconstruction. Of the 64 whole genomes from the literature, all were collected from domestic pig samples, except 7 had unknown host types (Supplementary Table S1).

**Figure 1:**
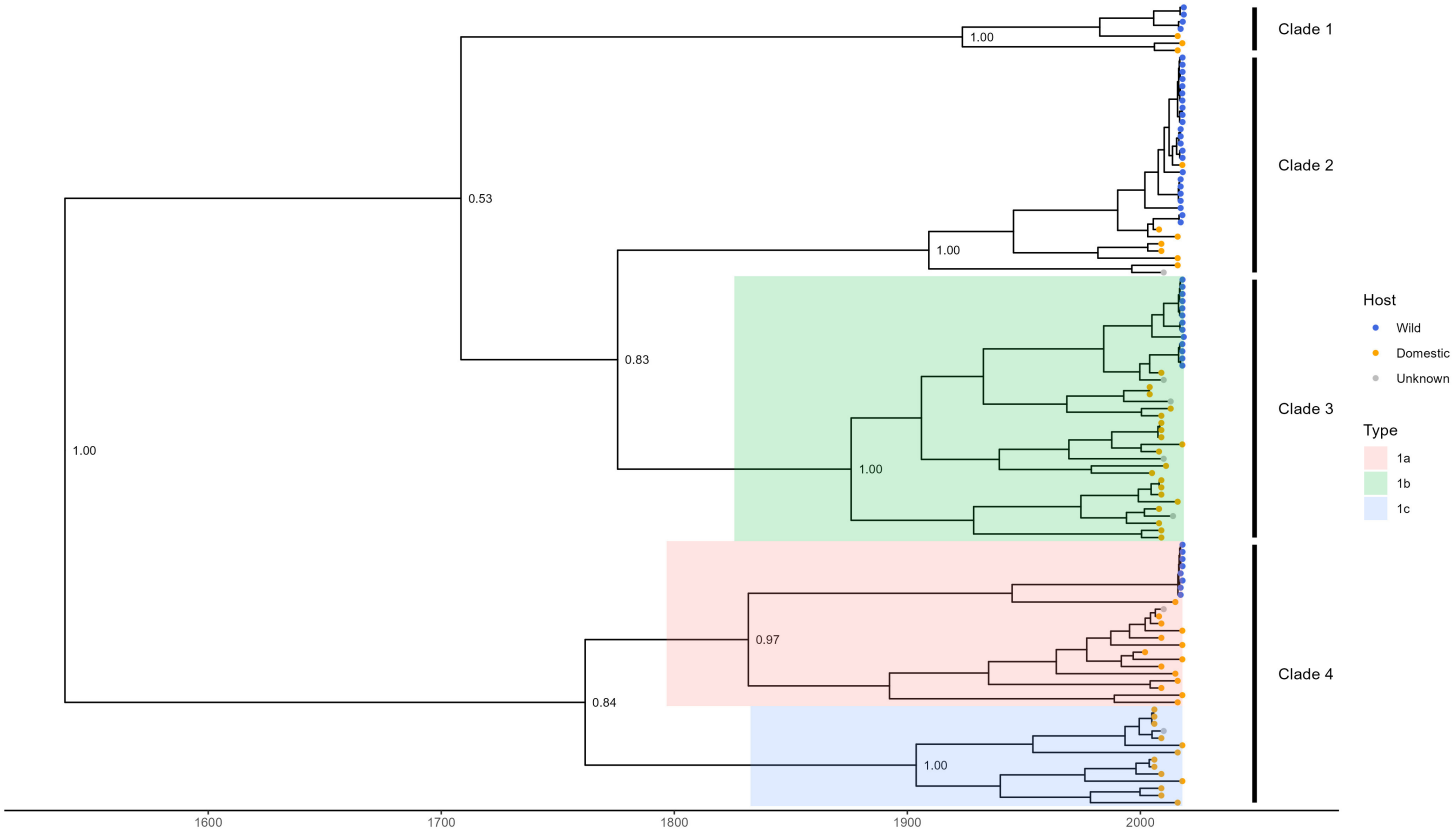
Time-dated phylogenetic tree of TTSuV1 whole genome sequences from this study and from NCBI. Blue tips in the tree represent sequences associated with wild pigs which are all from this study. Orange and gray tips represent sequences available in NCBI and associated with domestic pigs and unknown host, respectively. Shaded branches with colors represent different subtypes of TTSuV1 as defined by ICTV and other studies (Webb et al., 2020; Cortey et al., 2011; Huang et al., 2010; Liu et al., 2013). Clades were defined by the authors based on phylogenetic branching. For clarity, only bootstrap support values of major nodes are shown.

The dated phylogenetic tree grouped TTSuV1 whole genome sequences into four distinct clades (Figure 1). Sequences obtained from wild pigs in this study were distributed across all four clades and formed distinct branches that were generally separated from sequences associated with domestic pigs. Genetic classification of TTSuV1 based on whole genome sequences was less studied in the literature, and sequences corresponding to previously reported subtypes 1a, 1b, and 1c were clustered within Clades 3 and 4, leaving a number of sequences in Clades 1 and 2 with unidentified subtypes. The results highlight the extensive genetic variation of TTSuV1 genomes and suggest potential host-specific differentiation.

### 3.3 Genetic differentiation of TTSuV1 between host types

**Figure 2:**
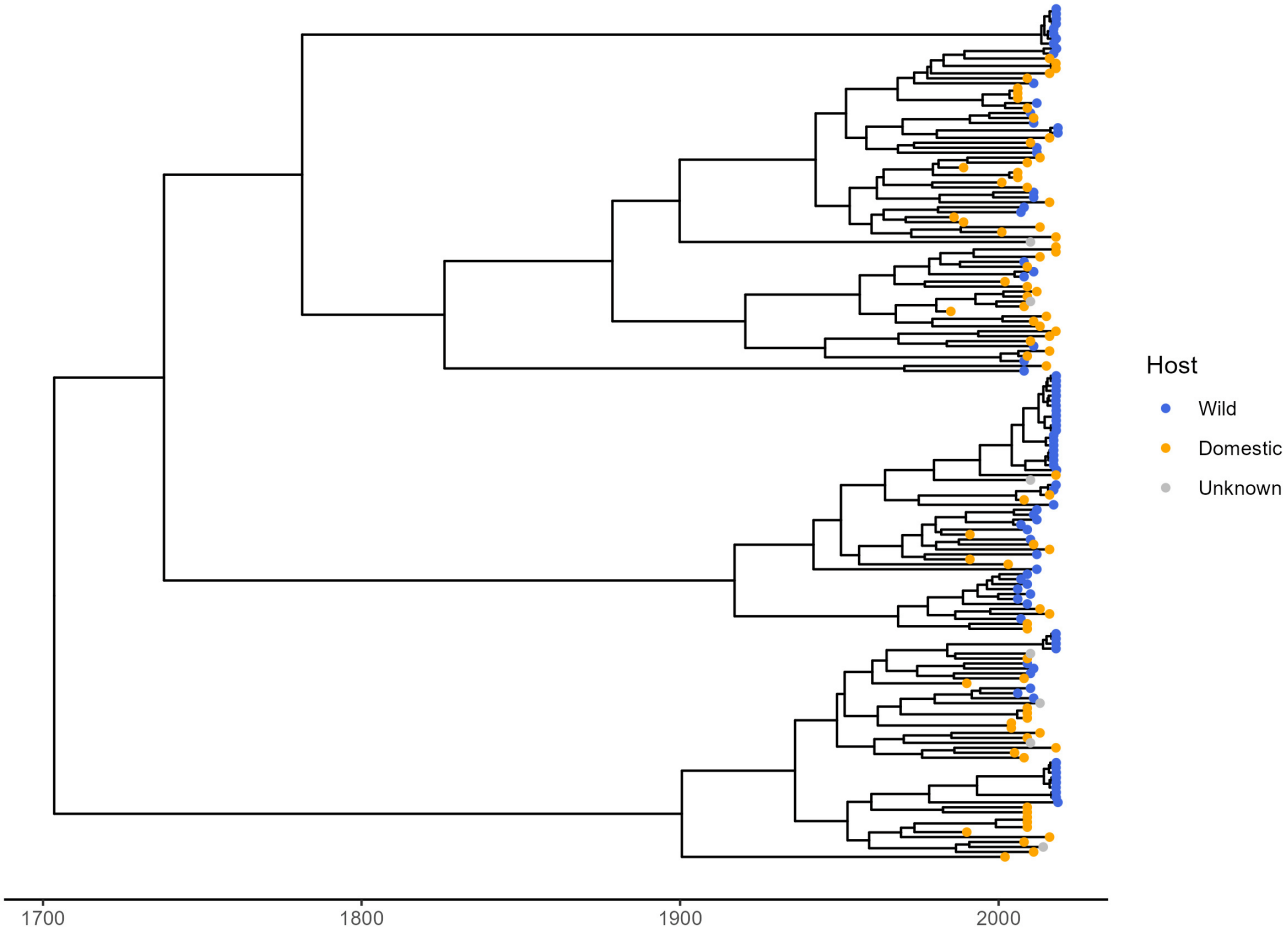
Time-dated phylogenetic tree of TTSuV1 partial sequences from this study and from NCBI. Blue and orange tips in the tree represent sequences associated with wild pigs and domestic pigs, respectively. Gray tips represent sequences with unknown host.

Through the literature search, 38 TTSuV1 partial sequences isolated from wild pigs were identified and downloaded from NCBI. These wild pig samples were collected from three countries: Spain, Romina, and Uruguay. From the same studies, 24 viral sequences from domestic pigs in these three countries were also obtained for this analysis, including the 110 trimmed whole genome sequences (2 sequences were excluded because of low coverage at 5'-UTR). A total of 172 TTSuV1 partial sequences were used to reconstruct the time-dated phylogenetic tree (Figure 2) and to determine genetic differentiation of the viral sequence between host types. Of them, 86 sequences came from wild pigs and 79 from domestic pigs globally.

The phylogenetic D statistic was calculated to assess the phylogenetic signal of the host type (wild vs. domestic) within the phylogenetic tree. The estimated D value was 0.32, closer to the expectation for Brownian (D = 0) phylogenetic structure and far from a random (D = 1) structure. The probability of the observed D value arising under a random phylogenetic structure was 0, while the probability of it arising under a Brownian motion model was 0.035. These results indicate a significant phylogenetic signal for host type, suggesting that the distribution of TTSuV1 in wild and domestic pigs is non-random and reflects some level of host-specific evolutionary differentiation.

The AMOVA revealed that most of the genetic variation (47.41%) was attributable to differences within host type, while 36.15% of the variation was explained by differences between countries (Table 1). A smaller proportion of variation (16.43%) was observed among host type within the same country. The Φ-statistics provided additional insights: the overall differentiation (Φ-samples-total = 0.53) showed that there was a significant genetic difference across all samples. The differentiation between countries (Φ-country-total = 0.36) indicated that geographic location played an important role in shaping genetic variation, while differentiation between wild and domestic pigs within countries (Φ-samples-country = 0.26) suggested that host type also contributed, though less strongly than geography.

**Table 1:** Genetic differentiation of TTSuV1 partial sequences based on analysis of molecular variance (AMOVA).

**Source of variation**	**Variance component (Sigma)**	**Percentage of variation (%)**	***p*-value**
Between countries	32.09	36.15	0.02
Between host types within country	14.59	16.43	0.01
Within host types	42.08	47.41	0.01
Total	88.76	100.00	

The randomization test confirmed the significance of these patterns. Genetic variation within host type was significantly smaller than expected by chance (*p* = 0.01), while variation between host types (*p* = 0.01) and between countries (*p* = 0.02) were significantly greater than random expectations. These results emphasize the importance of both geographic location and host type in structuring TTSuV1 genetic diversity.

### 3.4 Intra-host variation of TTSuV1

We analyzed the intra-host variation of TTSuV1 in pig individuals with more than one viral clone in our dataset. Pairwise identity percentages were calculated for 40 clones derived from 14 individuals. The majority of clone pairs from the same individual exhibited high identity, ranging from 99.3% to 100%. However, four clone pairs showed significantly lower identity percentages, with values ranging from 63.6% to 97% (Table 2). One pair of clones (453-4 and 453-2) were located in different branches within the same clade, while the rest of the three pairs had clones distributed in distinct clades. These results highlight that while most viral clones within a single host are highly similar, notable genetic divergence can occasionally occur, indicating co-infection with divergent viral strains.

**Table 2:** Identity percentage matrix of four pairs of distinctive (identity <99%) TTSuV1 clones. Samples from individual pigs 655, 674 and 732 had viral variants from separate clades.

	453-4	655-4	674-5	732-2
453-2	97			
655-1		63.6		
674-4			70.4	
732-1				70.1

Clones 453-4 and 453-2 were within the same clade but in different branches.

**Figure 3:**
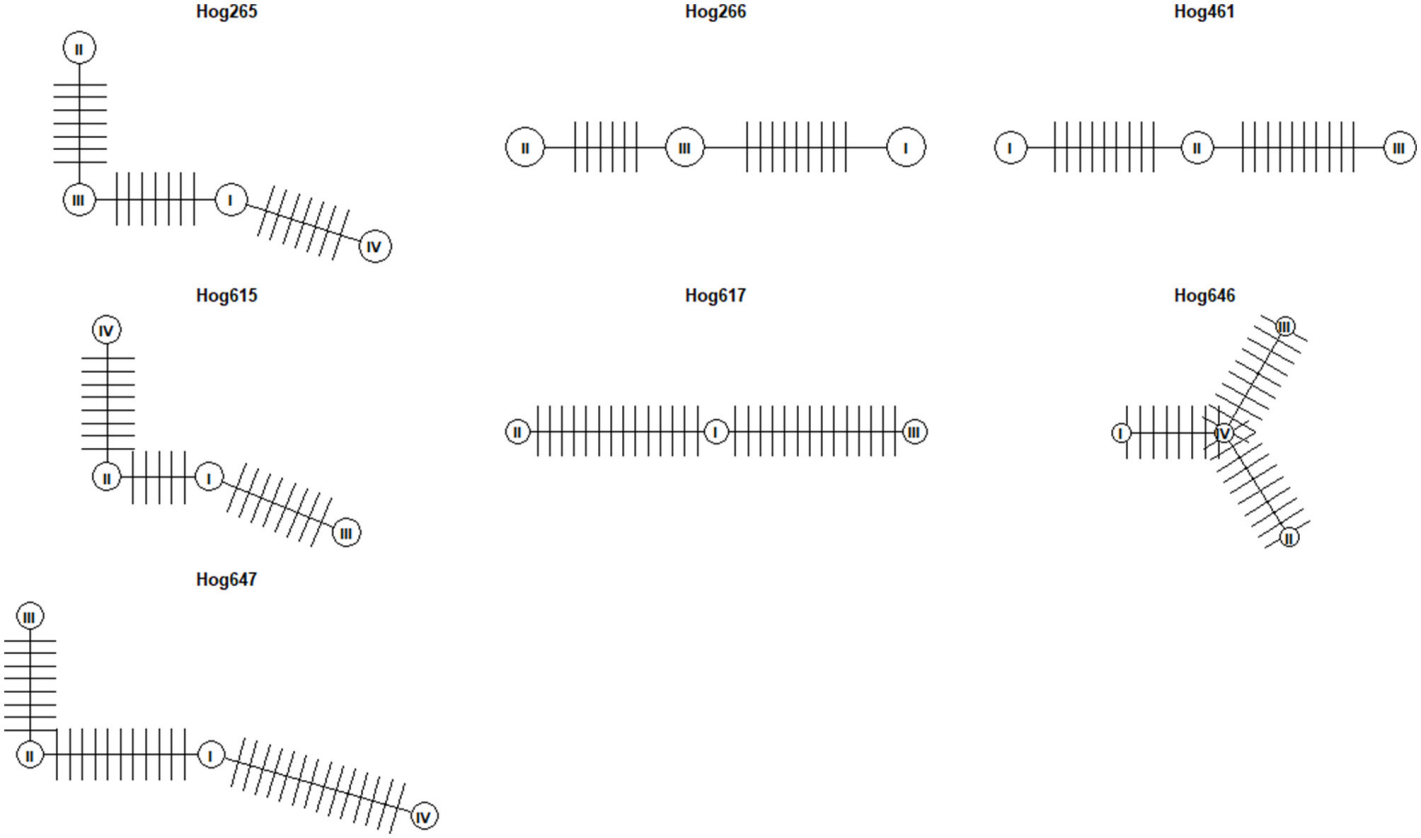
Haplotype networks of TTSuV1 clones from seven wild pigs. Each circle represents a distinct haplotype. Vertical bars between pairs of haplotypes indicate the number of mutational steps (single-nucleotide changes) separating them.

Haplotype network analysis was conducted on viral clones from seven wild pigs in this study, each with more than two clones, after excluding one pig (hog732) whose clones spanned across different phylogenetic clades. A haplotype network was constructed for each pig, revealing that the number of mutational steps, representing single-nucleotide changes separating one haplotype from another, within each network ranged from 6 to 14 (Figure 3). Since all clones from the same individual were confined to the same clade, the observed genetic variation likely resulted from intra-host evolution. These findings highlight the mutational dynamics shaping TTSuV1 diversity within individual hosts.

## 4 Discussion

In this study, we examined the genetic diversity, host-specific differentiation, and intra-host variation of Torque teno sus virus 1 (TTSuV1) using whole genome sequences and partial sequences derived from both wild and domestic pigs. Our findings revealed the presence of four distinct phylogenetic clades based on whole genomes, with sequences from wild pigs distributed across all clades, forming branches generally distinct from those of domestic pigs. Phylogenetic signal analysis using partial sequences confirmed a non-random association between host type and phylogeny, suggesting host-specific evolutionary differentiation. AMOVA further highlighted the contributions of both host type and geographic location to TTSuV1 genetic structure. Additionally, intra-host variation analysis demonstrated substantial genetic diversity among viral clones within individual pigs that suggested both co-infection of different variants of TTSuV1 and intra-host evolution as primary sources of diversity. These findings deepen our understanding of TTSuV1's evolutionary dynamics and its potential role in viral transmission and biosecurity risks.

### 4.1 Genetic diversity and phylogenetic clustering

Our phylogenetic analysis grouped TTSuV1 whole genome sequences into four distinct clades, illustrating the virus's extensive genetic diversity. Sequences obtained from wild pigs in this study were present in all four clades, forming separate branches from domestic pig sequences. Known subtypes 1a, 1b, and 1c were localized within Clades 3 and 4, but the broader clade structure observed here suggests additional diversity that is not captured by the current ICTV classification. This divergence is particularly notable for sequences in Clades 1 and 2, which may represent additional subtypes that have not yet been formally recognized.

These findings highlight the need for expanding TTSuV1 classification to incorporate whole genome data, moving beyond reliance on partial sequences. Whole genome data could provide a more comprehensive view of genetic variation, including regions that may be critical for understanding viral adaptation, host specificity, and transmission (Thami et al., 2023; Houldcroft et al., 2017; Wohl et al., 2016). By leveraging complete genomes, it becomes possible to identify novel subtypes, refine existing classifications, and establish robust phylogenetic relationships that reflect the true genetic complexity of TTSuV1. Whole genome data will also aid in downstream epidemiological applications if informative partial sequences can be identified.

Such an improvement would have significant implications for epidemiological studies. A well-resolved classification system based on whole genomes can serve as a reference for tracking the emergence and spread of distinct viral lineages across geographic regions and host populations (Goya et al., 2024; Hill et al., 2024). It would enhance the accuracy of phylogenetic analyses, allowing researchers to detect subtle evolutionary trends and identify factors driving viral diversification.

### 4.2 Host-specific differentiation

Genetic differentiation between TTSuV1 populations infecting wild and domestic pigs was assessed using partial sequences and evaluated through both phylogenetic signal analysis and AMOVA. The D statistic from the phylogenetic signal analysis demonstrated a significant non-random association between host type and phylogenetic structure, with viral sequences clustering by host type. This finding indicated that TTSuV1 evolution was influenced by host-specific factors, such as immune responses or ecological differences. A previous codon usage analysis based on the ORF1 gene assessed the host-specific adaptation of TTSuV species and revealed that TTSuV1 was more adapted to *Sus scrofa* than to *Sus scrofa domestica* (Li et al., 2019). Relative codon deoptimization index (RCDI) analysis suggested that TTSuV1 may replicate more efficiently or experience higher expression levels in wild pigs, reflecting a closer adaptation to this host. A similarity index (SiD) showed that wild pigs exert stronger selective pressures on TTSuV1 compared to domestic pigs. Thus, wild pigs play a significant role in shaping the evolutionary dynamics of TTSuV1, highlighting the impact of host-specific factors on the virus's adaptation and evolution.

AMOVA results provided further evidence of host-specific differentiation, with 16.43% of the genetic variation attributed to differences between host types within the same country. We also found a substantial proportion of the variance (36.15%) was explained by geographic differences between countries. These results align with studies on other swine viruses, such as porcine circovirus 2 (PCV2), which also identified both host and geographic factors as key contributors to genetic structure (Firth et al., 2009; Correa-Fiz et al., 2018). The randomization tests further validated these patterns; genetic variation within host types was significantly lower than expected under random conditions, and variation between host types and countries was significantly higher. These results emphasize the combined influence of host type and geographic separation on TTSuV1's genetic structure, reinforcing the importance of considering both factors in studies of viral transmission dynamics.

The presence of distinct clades and the separation of wild and domestic pig sequences suggest that the virus is undergoing host-specific adaptation, potentially influenced by ecological and immunological pressures. These evolutionary dynamics may have implications for viral transmission and persistence, particularly in regions where wild and domestic pigs interact.

### 4.3 Intra-host variation

The analysis of intra-host variation revealed considerable genetic differences among viral clones from the same individual. Pairwise identity percentages for most clones ranged from 99.3% to 100%, consistent with the high similarity expected from within host evolutionary processes. However, a small subset of clones exhibited significantly lower identity, with values as low as 63.6% which represented viral clones from different TTSuV subtypes and clades within the same host. These results suggest that intra-host variation can sometimes encompass highly divergent viral strains, resulting from co-infection with multiple TTSuV1 variants.

Haplotype networks constructed for seven pigs further illustrated intra-host genetic diversity. The networks showed mutational steps ranging from 6 to 14 bp changes between viral sequences, indicating gradual accumulation of genetic changes within the host. All clones with >99% identity within an individual belonged to the same phylogenetic clade, suggesting that intra-host variation also arises from intra-host evolution (e.g., mutations during replication). These results align with previous studies on other anelloviruses, such as human torque teno virus (TTV), where intra-host evolution has similarly been identified as a key driver of diversity (Kaczorowska et al., 2023; Laubscher et al., 2022).

The ability of TTSuV1 to generate substantial genetic diversity within hosts has implications for its persistence and adaptability. Rapid accumulation of mutations could enable the virus to evade immune responses or adapt to specific host environments, contributing to its success as a chronic infection. For example, Hepatitis C virus (HCV) exhibits a high mutation rate due to its RNA-dependent RNA polymerase lacking proofreading capability. This leads to the generation of diverse viral quasispecies, allowing it to evade immune recognition and persist as a chronic infection (Ortega-Prieto and Dorner, 2017). Although the quasispecies dynamics are commonly observed in RNA viruses, some DNA viruses, such as hepatitis B virus (HBV), also display quasispecies-like behavior, largely due to error-prone replication mechanisms and immune-driven selection pressures (Sardanyés et al., 2024). These patterns observed in other DNA viruses highlight the potential for TTSuV1 to maintain diverse intra-host populations that may contribute to its adaptability and long-term persistence. Understanding the sources and implications of intra-host variation is critical for unraveling TTSuV1's evolutionary strategies and its potential role in co-infections.

### 4.4 Implications for biosecurity monitoring

While there are advanced biosecurity monitoring systems in place for pig farms, most existing systems focus on internal biosecurity, pathogen tracing, and general risk assessments (Racicot et al., 2022; Bernaerdt et al., 2023; Scollo et al., 2022). Specific systems that monitor cross-transmission between wild and domestic pig populations remain largely undeveloped. The phylogenetic differentiation observed in this study, with distinct clustering of wild and domestic pig sequences, could serve as the foundation for a more integrated approach to biosecurity monitoring.

To effectively assess the risks of cross-transmission, it would be necessary to combine genetic differentiation analysis with existing biosecurity monitoring systems. Such an integrated approach could include the use of phylogenetic clustering to identify potential transmission events, genetic analysis to determine the relationships between wild and domestic pig populations, and applying these genetic insights to assess potential risks and further evaluate the effectiveness of current biosecurity strategies. This would allow for the detection of viral mixing between wild and domestic pigs, providing an early warning system to identify breaches and mitigate potential outbreaks.

Unlike highly pathogenic swine viruses, TTSuV1's non-pathogenic nature makes it an ideal candidate for biosecurity surveillance, as its presence is less likely to cause direct harm to swine while still reflecting transmission pathways of the virus (Li et al., 2024). With widespread distribution and high genetic diversity, TTSuV1 could serve as a robust genetic marker for detecting potential breaches in biosecurity or identifying cross-population hotspots at the wildlife-livestock interface, which will greatly contribute to biosecurity management of pig farms and disease management of emerging foreign animal diseases introduced by wild pig populations.

## 5 Limitations and future directions

While this study provides valuable insights into TTSuV1 diversity and evolution, several limitations should be acknowledged. First, the limited availability of whole genome sequences from wild pigs constrained our ability to fully characterize the genetic differences between wild and domestic populations. Expanding the dataset to include more sequences from diverse geographic regions and host types will be essential for a comprehensive understanding of TTSuV1 epidemiology.

Second, the reliance on partial sequences for some analyses, while necessary to overcome data limitations, provided a less complete picture of genetic variation than whole genome data. Future studies should aim to increase the use of WGS to refine subtype classification and explore the full spectrum of TTSuV1 genetic diversity.

Additionally, while genetic differentiation can indicate viral transmission between wild and domestic pigs, it does not reveal specific transmission pathways or the role of fomites in viral spread. Furthermore, the stability of TTSuV1 in the environment is unstudied, yet critical if TTSuV1 is to be used for evaluating biosecurity practices. Future studies on viral shedding, stability, and transmission dynamics are needed to enhance the interpretation of genetic data in biosecurity applications.

Lastly, while our results suggest that intra-host evolution and co-infection of different TTSuV1 variants are key drivers of genetic variation, our analysis of within host viral variant diversity using TOPO TA cloning was rudimentary. While it provided concrete evidence of within host diversity, it likely grossly underestimated the level and nature of diversity within hosts. Future studies should use next generation technologies to further develop this line of inquiry. Advanced molecular analyses could, for example, help better understand the role of recombination in generating diversity. High-resolution sequencing of viral populations within individual hosts, coupled with experimental studies, could help disentangle these processes and their contributions to TTSuV1 evolution.

## 6 Conclusions

This study highlights the extensive genetic differentiation and intra-host variation of TTSuV1, providing a deeper understanding of its evolutionary dynamics. Phylogenetic and AMOVA analyses showed that both host type and geographic location influence TTSuV1's genetic structure, with wild and domestic pig sequences forming distinct clusters. Intra-host variation analyses demonstrated that both intra-host evolution and co-infection with distinct variants contribute to genetic diversity within individual hosts.

These findings underscore the importance of integrating whole genome sequencing and phylogenetic analyses to explore the genetic structure of TTSuV1. Understanding the factors shaping viral diversity and transmission will be critical for monitoring cross-population transmission, improving biosecurity measures, and managing the risks at the wildlife-livestock interface. As TTSuV1 research progresses, expanding genomic datasets and incorporating advanced analytical methods will be essential for uncovering the full complexity of this virus's ecology and evolution.

## Data Availability

The datasets presented in this study can be found in online repositories. The names of the repository/repositories and accession number(s) can be found in the article/Supplementary material.
