# Supplementary material for: Whole genome characterization of Torque teno sus virus 1 (TTSuV1) in wild and domestic pigs: insights into genetic classification, host differentiation, and intra-host variation
Source: Front Microbiol. 2025 May 9;16:1585558. doi: 10.3389/fmicb.2025.1585558 (PMC12098577; doi:10.3389/fmicb.2025.1585558)
Supplement: Supplementary file 1 [file Table_1.docx]

Table S1. Partial and whole genome sequences of Torque teno sus virus 1 used in this study.

| Accession No. | Host | Country | Sequence Type | Source |
| --- | --- | --- | --- | --- |
| PV068559 | Wild | USA | Whole genome | This study |
| PV068560 | Wild | USA | Whole genome | This study |
| PV068561 | Wild | USA | Whole genome | This study |
| PV068562 | Wild | USA | Whole genome | This study |
| PV068563 | Wild | USA | Whole genome | This study |
| PV068564 | Wild | USA | Whole genome | This study |
| PV068565 | Wild | USA | Whole genome | This study |
| PV068566 | Wild | USA | Whole genome | This study |
| PV068567 | Wild | USA | Whole genome | This study |
| PV068568 | Wild | USA | Whole genome | This study |
| PV068569 | Wild | USA | Whole genome | This study |
| PV068570 | Wild | USA | Whole genome | This study |
| PV068571 | Wild | USA | Whole genome | This study |
| PV068572 | Wild | USA | Whole genome | This study |
| PV068573 | Wild | USA | Whole genome | This study |
| PV068574 | Wild | USA | Whole genome | This study |
| PV068575 | Wild | USA | Whole genome | This study |
| PV068576 | Wild | USA | Whole genome | This study |
| PV068577 | Wild | USA | Whole genome | This study |
| PV068578 | Wild | USA | Whole genome | This study |
| PV068579 | Wild | USA | Whole genome | This study |
| PV068580 | Wild | USA | Whole genome | This study |
| PV068581 | Wild | USA | Whole genome | This study |
| PV068582 | Wild | USA | Whole genome | This study |
| PV068583 | Wild | USA | Whole genome | This study |
| PV068584 | Wild | USA | Whole genome | This study |
| PV068585 | Wild | USA | Whole genome | This study |
| PV068586 | Wild | USA | Whole genome | This study |
| PV068587 | Wild | USA | Whole genome | This study |
| PV068588 | Wild | USA | Whole genome | This study |
| PV068589 | Wild | USA | Whole genome | This study |
| PV068590 | Wild | USA | Whole genome | This study |
| PV068591 | Wild | USA | Whole genome | This study |
| PV068592 | Wild | USA | Whole genome | This study |
| PV068593 | Wild | USA | Whole genome | This study |
| PV068594 | Wild | USA | Whole genome | This study |
| PV068595 | Wild | USA | Whole genome | This study |
| PV068596 | Wild | USA | Whole genome | This study |
| PV068597 | Wild | USA | Whole genome | This study |
| PV068598 | Wild | USA | Whole genome | This study |
| PV068599 | Wild | USA | Whole genome | This study |
| PV068600 | Wild | USA | Whole genome | This study |
| PV068601 | Wild | USA | Whole genome | This study |
| PV068602 | Wild | USA | Whole genome | This study |
| PV068603 | Wild | USA | Whole genome | This study |
| PV068604 | Wild | USA | Whole genome | This study |
| PV068605 | Wild | USA | Whole genome | This study |
| PV068606 | Wild | USA | Whole genome | This study |
| ON131018 | Domestic | Switzerland | Whole genome | NCBI |
| MW051669 | Domestic | USA | Whole genome | NCBI |
| MK990454 | Domestic | Canada | Whole genome | NCBI |
| MW117138 | Domestic | China | Whole genome | NCBI |
| MT671998 | Domestic | Brazil | Whole genome | NCBI |
| MT671997 | Domestic | Brazil | Whole genome | NCBI |
| MT671996 | Domestic | Brazil | Whole genome | NCBI |
| MT671969 | Domestic | Brazil | Whole genome | NCBI |
| MT671968 | Domestic | Brazil | Whole genome | NCBI |
| MT671967 | Domestic | Brazil | Whole genome | NCBI |
| MN272072 | Domestic | Brazil | Whole genome | NCBI |
| MH170071 | Domestic | Brazil | Whole genome | NCBI |
| MH170070 | Domestic | Brazil | Whole genome | NCBI |
| MH170069 | Domestic | Brazil | Whole genome | NCBI |
| MH170068 | Domestic | Brazil | Whole genome | NCBI |
| MH170067 | Domestic | Brazil | Whole genome | NCBI |
| MH170066 | Domestic | Brazil | Whole genome | NCBI |
| MH170065 | Domestic | Brazil | Whole genome | NCBI |
| MH170064 | Domestic | Brazil | Whole genome | NCBI |
| MH170063 | Domestic | Brazil | Whole genome | NCBI |
| MH170062 | Domestic | Brazil | Whole genome | NCBI |
| NC_027059 | Domestic | Brazil | Whole genome | NCBI |
| NC_014070 | Domestic | Japan | Whole genome | NCBI |
| KY742733 | Domestic | Brazil | Whole genome | NCBI |
| KT037083 | Domestic | USA | Whole genome | NCBI |
| KT968712 | Unknown | China | Whole genome | NCBI |
| KR054745 | Domestic | Japan | Whole genome | NCBI |
| KT174486 | Unknown | China | Whole genome | NCBI |
| AY823990 | Domestic | Brazil | Whole genome | NCBI |
| JQ933527 | Domestic | China | Whole genome | NCBI |
| JX535327 | Unknown | China | Whole genome | NCBI |
| JX535326 | Unknown | China | Whole genome | NCBI |
| JX535325 | Unknown | China | Whole genome | NCBI |
| JF937662 | Domestic | China | Whole genome | NCBI |
| JF937661 | Domestic | China | Whole genome | NCBI |
| JF937660 | Domestic | China | Whole genome | NCBI |
| JF694117 | Unknown | China | Whole genome | NCBI |
| JF694116 | Unknown | China | Whole genome | NCBI |
| GU570202 | Domestic | Spain | Whole genome | NCBI |
| GU570201 | Domestic | Spain | Whole genome | NCBI |
| GU570200 | Domestic | Spain | Whole genome | NCBI |
| GU570199 | Domestic | Spain | Whole genome | NCBI |
| GU570198 | Domestic | Spain | Whole genome | NCBI |
| GQ120664 | Domestic | Canada | Whole genome | NCBI |
| GU456384 | Domestic | USA | Whole genome | NCBI |
| GU456383 | Domestic | USA | Whole genome | NCBI |
| GU188045 | Domestic | Germany | Whole genome | NCBI |
| HM633258 | Domestic | China | Whole genome | NCBI |
| HM633257 | Domestic | China | Whole genome | NCBI |
| HM633256 | Domestic | China | Whole genome | NCBI |
| HM633255 | Domestic | China | Whole genome | NCBI |
| HM633254 | Domestic | China | Whole genome | NCBI |
| HM633253 | Domestic | China | Whole genome | NCBI |
| HM633252 | Domestic | China | Whole genome | NCBI |
| HM633251 | Domestic | China | Whole genome | NCBI |
| HM633250 | Domestic | China | Whole genome | NCBI |
| HM633249 | Domestic | China | Whole genome | NCBI |
| HM633248 | Domestic | China | Whole genome | NCBI |
| HM633247 | Domestic | China | Whole genome | NCBI |
| HM633246 | Domestic | China | Whole genome | NCBI |
| HM633245 | Domestic | China | Whole genome | NCBI |
| HM633244 | Domestic | China | Whole genome | NCBI |
| HM633243 | Domestic | China | Whole genome | NCBI |
| HM633242 | Domestic | China | Whole genome | NCBI |
| GU456383.1 | Domestic | USA | Partial sequence | NCBI |
| GU456384.1 | Domestic | USA | Partial sequence | NCBI |
| GU570198.1 | Domestic | Spain | Partial sequence | NCBI |
| GU570199.1 | Domestic | Spain | Partial sequence | NCBI |
| GU570200.1 | Domestic | Spain | Partial sequence | NCBI |
| GU570201.1 | Domestic | Spain | Partial sequence | NCBI |
| GU570202.1 | Domestic | Spain | Partial sequence | NCBI |
| JF451459.1 | Domestic | Spain | Partial sequence | NCBI |
| JF451474.1 | Domestic | Spain | Partial sequence | NCBI |
| JF451491.1 | Domestic | Spain | Partial sequence | NCBI |
| JF451499.1 | Domestic | Spain | Partial sequence | NCBI |
| JF451500.1 | Domestic | Spain | Partial sequence | NCBI |
| JF451539.1 | Domestic | Spain | Partial sequence | NCBI |
| JF451549.1 | Domestic | Spain | Partial sequence | NCBI |
| JF451550.1 | Domestic | Spain | Partial sequence | NCBI |
| JF451568.1 | Domestic | Spain | Partial sequence | NCBI |
| JF451571.1 | Domestic | Spain | Partial sequence | NCBI |
| JF451575.1 | Domestic | Spain | Partial sequence | NCBI |
| JF451577.1 | Domestic | Spain | Partial sequence | NCBI |
| JN181957.1 | Wild | Spain | Partial sequence | NCBI |
| JN181970.1 | Wild | Spain | Partial sequence | NCBI |
| JN181971.1 | Wild | Spain | Partial sequence | NCBI |
| JN181979.1 | Wild | Spain | Partial sequence | NCBI |
| JX444412.1 | Domestic | Romania | Partial sequence | NCBI |
| JX444413.1 | Domestic | Romania | Partial sequence | NCBI |
| JX444414.1 | Domestic | Romania | Partial sequence | NCBI |
| JX444415.1 | Wild | Romania | Partial sequence | NCBI |
| JX444416.1 | Wild | Romania | Partial sequence | NCBI |
| JX444417.1 | Wild | Romania | Partial sequence | NCBI |
| JX444418.1 | Wild | Romania | Partial sequence | NCBI |
| JX444419.1 | Wild | Romania | Partial sequence | NCBI |
| JX444420.1 | Wild | Romania | Partial sequence | NCBI |
| JX444421.1 | Wild | Romania | Partial sequence | NCBI |
| JX444422.1 | Wild | Romania | Partial sequence | NCBI |
| JX444423.1 | Wild | Romania | Partial sequence | NCBI |
| JX444424.1 | Wild | Romania | Partial sequence | NCBI |
| JX444425.1 | Wild | Romania | Partial sequence | NCBI |
| JX444426.1 | Wild | Romania | Partial sequence | NCBI |
| JX444427.1 | Wild | Romania | Partial sequence | NCBI |
| JX444428.1 | Wild | Romania | Partial sequence | NCBI |
| JX444429.1 | Wild | Romania | Partial sequence | NCBI |
| JX444430.1 | Wild | Romania | Partial sequence | NCBI |
| JX444431.1 | Wild | Romania | Partial sequence | NCBI |
| JX444432.1 | Wild | Romania | Partial sequence | NCBI |
| JX444433.1 | Wild | Romania | Partial sequence | NCBI |
| JX444434.1 | Wild | Romania | Partial sequence | NCBI |
| JX444435.1 | Wild | Romania | Partial sequence | NCBI |
| JX444436.1 | Wild | Romania | Partial sequence | NCBI |
| JX444437.1 | Wild | Romania | Partial sequence | NCBI |
| JX444438.1 | Wild | Romania | Partial sequence | NCBI |
| JX444439.1 | Wild | Romania | Partial sequence | NCBI |
| JX444440.1 | Wild | Romania | Partial sequence | NCBI |
| JX444441.1 | Wild | Romania | Partial sequence | NCBI |
| JX444442.1 | Wild | Romania | Partial sequence | NCBI |
| JX444443.1 | Wild | Romania | Partial sequence | NCBI |
| JX444444.1 | Wild | Romania | Partial sequence | NCBI |
| JX444445.1 | Wild | Romania | Partial sequence | NCBI |
| JX444446.1 | Wild | Romania | Partial sequence | NCBI |
| MH469964.1 | Domestic | Uruguay | Partial sequence | NCBI |
| MH469965.1 | Domestic | Uruguay | Partial sequence | NCBI |
| MH469966.1 | Wild | Uruguay | Partial sequence | NCBI |
| MH469967.1 | Domestic | Uruguay | Partial sequence | NCBI |
| MH469968.1 | Wild | Uruguay | Partial sequence | NCBI |
| MH469970.1 | Domestic | Uruguay | Partial sequence | NCBI |
| MH469971.1 | Domestic | Uruguay | Partial sequence | NCBI |
| MH469972.1 | Domestic | Uruguay | Partial sequence | NCBI |
| MH469973.1 | Domestic | Uruguay | Partial sequence | NCBI |
| MH469974.1 | Domestic | Uruguay | Partial sequence | NCBI |
| MH469975.1 | Domestic | Uruguay | Partial sequence | NCBI |
| JF451557.1 | Domestic | USA | Partial sequence | NCBI |
| JF451556.1 | Domestic | USA | Partial sequence | NCBI |
| JF451489.1 | Domestic | USA | Partial sequence | NCBI |
| JF451462.1 | Domestic | USA | Partial sequence | NCBI |
| JF451447.1 | Domestic | USA | Partial sequence | NCBI |
